# Increased Insulin following an Oral Glucose Load, Genetic Variation near the Melatonin Receptor *MTNR1B*, but No Biochemical Evidence of Endothelial Dysfunction in Young Asian Men and Women

**DOI:** 10.1371/journal.pone.0133611

**Published:** 2015-07-21

**Authors:** Maria A. Matuszek, Angelyn Anton, Sobana Thillainathan, Nicola J. Armstrong

**Affiliations:** 1 School of Medical Sciences, University of New South Wales, Sydney, Australia; 2 Mathematics and Statistics, Murdoch University, Perth, Australia; University of Brescia, ITALY

## Abstract

**Aim:**

To identify biochemical and genetic variation relating to increased risk of developing type 2 diabetes mellitus and cardiovascular disease in young, lean male and female adults of different ethnicities.

**Method:**

Fasting blood and urine and non-fasting blood following oral glucose intake were analysed in 90 Caucasians, South Asians and South East/East Asians.

**Results:**

There were no differences in age, birthweight, blood pressure, body mass index, percent body fat, total energy, percentage of macronutrient intake, microalbumin, leptin, cortisol, adrenocorticotropic hormone, nitric oxide metabolites, C-reactive protein, homocysteine, tumor necrosis factor-α, interleukin-6, von Willebrand factor, vascular cell adhesion molecule-1, plasminogen activator inhibitor-1, and tissue plasminogen activator. Fasting total cholesterol (P = .000), triglycerides (P = .050), low density lipoprotein (P = .009) and non-fasting blood glucose (15 min) (P = .024) were elevated in South Asians compared with Caucasians, but there was no significant difference in glucose area under curve (AUC). Non-fasting insulin in South Asians (15–120 min), in South East/East Asians (60–120 min), and insulin AUC in South Asians and South East/East Asians, were elevated compared with Caucasians (P≤0.006). The molar ratio of C-peptide AUC/Insulin AUC (P = .045) and adiponectin (P = .037) were lower in South Asians compared with Caucasians. A significant difference in allele frequency distributions in Caucasians and South Asians was found for rs2166706 (P = 0.022) and rs10830963 (P = 0.009), which are both near the melatonin receptor *MTNR1B*.

**Conclusions:**

Elevated non-fasting insulin exists in young South Asians of normal fasting glucose and insulin. Hepatic clearance of insulin may be reduced in South Asians. No current biochemical evidence exists of endothelial dysfunction at this stage of development. *MTNR1B* signalling may be a useful therapeutic target in Asian populations in the prevention of type 2 diabetes mellitus.

## Introduction

The metabolic syndrome, a clustering of cardiometabolic risk factors (abdominal obesity, hyperglycaemia, dyslipidaemia, hypertension), increases an individual’s probability of developing type 2 diabetes mellitus (T2DM) or cardiovascular disease, and varies significantly among ethnic groups [1]. Insulin resistance (IR) and T2DM has increased in countries which have adopted a ‘western lifestyle’ (comprised of reduced physical activity and a diet higher in fat) with some ethnic groups having a higher prevalence of this disease compared to other groups living in the same multiethnic environment. It is more common in peoples of non-Caucasian compared to Caucasian (C) origin and most wide spread in Asia/Australasia with 82.7 million diagnosed, which is half of the world-wide prevalence. [1, 2]. South-Asians (SA), especially Asian-Indians appear to be the most insulin resistant. Compared with a 5% incidence in C, the prevalence of T2DM in Asian-Indians living in ‘westernised’ countries is around 19% and develops about 10 years earlier. South-East Asians are also highly susceptible with an estimated 8% and 12% incidence in peoples from Malaysia or Thailand, respectively [3].

Many studies have focused on older (>40 years), overweight participants with well established IR, however it is evident that IR is becoming increasingly prevalent in youth. A previous study [4] observed that younger (18–35 year old), leaner (body mass index (BMI) < 25 kg/m^2^) adults without T2DM, already have elevated glucose and insulin following an oral glucose load. This effect was marked in the Asians compared to C. That study, however, did not examine for other blood markers of the metabolic syndrome.

High blood insulin and glucose are damaging to blood vessel function thus T2DM is considered a risk factor for cardiovascular disease, with 60–80% of people with diabetes having hypertension and around 75% of deaths in this population due to cardiovascular disease [5]. Conversely, many cardiovascular risk factors are present before the development of T2DM. One similarity between the two is endothelial dysfunction (ED) [6]. The term refers to impaired vasodilation to specific mediators and to a proinflammatory and prothrombic state associated with the vascular endothelium [7]. It appears to precede the development of T2DM or cardiovascular disease, and has been identified in young normotensive individuals without diabetes but with IR [8] and in young normotensive offspring whose parents have either hypertension [9] or diabetes [10]. A correlation between ED, and higher non-fasting glucose and insulin has been observed in individuals with a normal fasting glucose [10].

The current study aims were threefold, the first being a confirmation whether glucose and insulin following an oral glucose load are elevated in younger leaner individuals of Asian compared to Caucasian origin, despite a normal fasting glucose. If so, this would further emphasize that the oral glucose tolerance test (OGTT) which measures the rise and fall of blood glucose for 2 hr following a meal, together with the measurement of insulin, is a more suitable diagnostic than fasting blood glucose alone, in identifying young ‘at risk’ participants in populations in whom diabetes is more prevalent. Secondly, though an interrelationship between T2DM and cardiovascular disease, with ED as an early common denominator has been established, in older overweight populations [11], this study undertook for the first time an extensive biochemical screening for markers of ED in younger non-overweight, non-obese adults of different ethnicities in whom prediabetes is absent. Any biochemical markers successfully identified could serve as useful future diagnostic tools, along with contributing to existing knowledge on the early onset of ED, T2DM and cardiovascular complications. This study measured blood glucose, insulin, C-peptide, glycosylated haemoglobin (HbA1C), lipids (total cholesterol, triglycerides, low and high density lipoproteins), cortisol, adrenocorticotrophic hormone (ACTH), nitric oxide (NO) metabolites (nitrate, nitrite), the prothrombic markers (tissue plasminogen activator (t-PA), plasminogen-activator inhibitor-1 (PAI-1) and von Willebrand factor), the proinflammatory markers (C-reactive protein (CRP), homocysteine, interleukin-6 (IL-6) and tumor necrosis factor-α (TNF-α)), the soluble markers of inflammation (vascular cell adhesion molecule-1 (VCAM-1) and endothelial-leukocyte adhesion molecule-1 (E-selectin)), adipose tissue biomarkers (adiponectin and leptin), and urinary creatinine and microalbumin.

Thirdly, while the environmental factors of sedentary lifestyle and consumption of energy-dense foods undoubtedly contribute to T2DM, this condition also appears to have a genetic contribution. The World Health Organization (WHO) estimates that by 2025, one-quarter of T2DM patients globally will be Asian Indian [12]. Indians, within India and elsewhere, have the highest prevalence of T2DM, earning the unfortunate term of ‘diabetes capital of the world’ [12, 13]. Therefore, in addition to the above, the current study also examined both the Caucasian and Asian populations for single nucleotide polymorphism (SNP) frequencies in genes associated with T2DM risk [14]. The risk of T2DM and the metabolic syndrome is variable, even within the Asian population. Studies have separately grouped SA and South East Asians in their comparisons with other cohorts [4]. The International Diabetes Federation has also acknowledged differences within the Asian population by distinguishing between SA, Chinese and Japanese in their ethnic specific cut-off points for waist circumference [15]. Therefore this study kept separate the SA and the South East and East Asian (SEA) groups to examine for differences between them and C.

## Methods

### Study population

The study was approved by the University of New South Wales Human Research Ethics Committee (HREC 05311). All participants in the study provided a written consent. Ninety male and female volunteer university students (30 per group) of C, SA (Sri Lankan, Indian, Pakistani, Bangladeshi) and SEA (Vietnamese, Cambodian, Indonesian, Malaysian, Philippine, Burmese, Korean) ethnicity were recruited by a written advertisement distributed on notice boards, university publications and electronic university communications. They were admitted into the study after satisfying the following: age 18–25 years, BMI 18–25 kg/m², waist circumference < 90cm (males) and < 80 cm (females), and blood pressure <140/90 mmHg (since overweight and hypertension contribute to IR). Participants were excluded if they (via a questionnaire) had any known cardiovascular or non-cardiovascular disease, were on medication that could interfere with the study, were current smokers or had smoked within the last six months. A family history (parent(s) and/or grandparent(s)) of hypertension and/or T2DM was recorded and verified where possible with a list of medications. The following were also recorded: birth weight (as reported by their parents; low birth weight < 2.5 kg or high birth weight > 4 kg is linked to an increased risk of insulin resistance later in life) [16], exercise habit (to be aware of potential confounding beneficial effects of regular aerobic and resistance exercise) [17], and the phase of the menstrual cycle during which female participants undertook the OGTT (there are contradictions regarding the effect of the menstrual cycle on insulin sensitivity) [18–21].

### Study design

Participants made two visits to the laboratory.

#### Session 1

Heart rate and blood pressure using an automated device (Omron M2) were measured with the subject seated after 15 minutes of rest (mean of 3 readings). Anthropometric measurements used standardised techniques. Percent body fat was measured both by skin folds using the Durnin-Womersley equation [22] validated for Asian Indians [23], and a TANITA bioelectrical impedance analyser (Model BWB-800). TANITA has been well correlated with dual-energy X-ray absorptiometry (DEXA) and hydrodensitometry [24].

#### Session 2

Participants arrived in the morning after an overnight fast since 8 pm; and abstinence from alcohol, tea, coffee, caffeine-containing foods, and foods high in nitrites/nitrates in the previous 48 hr. As the act of venepuncture can increase some hormone levels by more than 50% [25], a stabilization period of 30 min was allowed after a cannula was inserted into a forearm vein and before blood collection. Fasting blood (33 ml) (for the measurement of all markers) was collected into vacutainers containing ethylenediamine tetracetic acid (EDTA) or trisodium citrate between 7–10 am with participants in a semi-inclined position. Following a 75-g glucose load, blood (each 6 ml) was collected every 15 min over 2 hr for the measurement of glucose, insulin and C-peptide. Glucose, lipids, and HbA1C were measured immediately. Remaining blood was centrifuged (1560 g, 10 min, Heraeus Megafuge, Germany) and plasma stored at -86°C for the measurement of other biomarkers. The buffy coat (0.5 ml) from fasting blood containing the leucocytes was stored at -86°C following stabilization in 1.2 ml of RNA*later* (Ambion, USA) for SNP frequency analysis. Microalbumin and creatinine were measured on the same day of collection from a mid-stream urine sample, collected upon rising or immediately prior to cannulation. Participants completed a 3-day food diary, indicating type and quantity of food consumed during two non-consecutive weekdays and one weekend day. As participants were requested to abstain from certain foods in the 48 hrs prior to the OGTT, nutritional intake during this period was not recorded in the food diary. Data were entered into a nutritional database (Serve Nutrition Systems, Australia).

### Biochemical and genetic analysis

Blood glucose (HemoCue, Sweden), lipids (Cholestech LDX, USA), HbA1C, microalbumin and creatinine (DCA 2000, Bayer HealthCare, USA) were measured by reflectance photometry. Plasma insulin (BioQuant, USA), C-peptide (Demeditec Diagnostics, Germany), CRP, cortisol, ACTH, leptin (each DSL, USA), homocysteine (Axis-Shield Diagnostics, UK), t-PA, PAI-1, IL-6, TNF-α, E-selectin, VCAM-1 (each Bender MedSystems, Austria), von Willebrand Factor (Corgenix, USA) and adiponectin (R+D Systems, USA) were determined by ELISA with colorimetric assay at 450 nm. For the indirect determination of NO, 400 ul of each plasma sample was ultrafiltered through VectaSpin micro-polysulphone 30kD MW cutoff filters (Whatman, UK) (4620 g, 60 min, 24°C, Hettich Zentrifugen EBA12R, Germany). Plasma nitrite was then determined at 550 nm using a colorimetric assay based on the Griess method (Cayman Chemical Company, USA). All colorimetric signals were measured using an Expert Plus Microplate reader (Asys Hitech, Austria). Both the extraction of DNA from fasting blood and SNP genotyping was performed by the Australian Genome Research Facility Ltd.

### Statistical analysis

#### Biochemistry

Homeostasis model assessment of insulin resistance (HOMA-IR) [26] assessed insulin sensitivity with the formula: fasting insulin (pmol/l) x fasting glucose (mmol/l)/22.5. For estimating insulin clearance, the molar ratios of the integrated AUC response of C-peptide (pmol/l) over insulin (pmol/l) after oral glucose were calculated [27].

Power calculations, based on a previous study [4], determined that 10 participants per group would be sufficient to detect a significant difference in area under curve (AUC) for glucose and insulin, assuming a 0.05 significance level and 80% power. Actual sample size was 30 per group (or 15 per group when studying gender differences). AUC were calculated using SigmaPlot 2001 (SPSS Inc., USA.) using the trapezoidal method. Glucose, insulin and C-peptide AUC, all other blood/urine biochemistry, physiological characteristics and diet were compared using one-way ANOVA. Post hoc comparisons were performed using the Tukey HSD test except for folate consumption when least square difference (LSD) method was used. Correlation was assessed using Pearson correlation. Data are presented as mean ± SEM. All tests were two-sided, and P<0.05 indicated statistical significance. Analysis was conducted in SPSS (SPSS Inc., U.S.A).

#### Genetics

Association analyses between SNPs and insulin AUC were run both ignoring or including ethnicity as a factor (either C vs SA and SEA or C vs SA vs SEA) using PLINK [28]. Allele frequency distributions in C and SA were compared using Fisher’s exact test in the R package (v3.0.1).

## Results

### Study population characteristics

The C, SA and SEA groups, each contained participants whose parent(s) and/or grandparent(s) were diagnosed as having diabetes (D), hypertension (H), or both diabetes/hypertension (DH). C consisted of 2D, 14H, 9DH; SA had 5D, 5H and 14DH, and SEA had 2D, 8H, and 13DH. The C, SA and SEA groups each had 5, 6 and 7 participants, respectively, reporting no family history for either of the conditions.

There was no significant difference in age or birth weight between groups. Of the 90 participants in the study, 77 participants had a normal birth weight of 2.5–4 kg. The birth weights of 3 C, 4 SA and 1 SEA were 4.2–4.8 kg. A C was 2.3 kg, one SA was 2.2 kg (premature at 32 weeks) and a SEA was 1.7 kg (a twin born at 36 weeks). One SA and SEA recorded their birth weight as unknown.

Regarding exercise habit, 26% of the group of 90 participants did not engage in physical activity, with 23 participants (5 C, 6 SA, 12 SEA) reporting by questionnaire that they were sedentary or did not participate in regular aerobic exercise or resistance training. A weekly duration of < 1 hour, 1–2 hours, and > 2hours of aerobic exercise (for example, running, cycling, rowing or swimming) was recorded for 39 (14 C, 13 SA, 12 SEA), 16 (5 C, 8 SA, 3 SEA) and 4 (3 C, 1 SEA) participants respectively. The 4 participants who indicated that they were engaged in > 2 hours/week of aerobic exercise had average resting heart rates of 77–89 bpm. Resistance training of < 1 hour, 1–2 hours, and > 2 hours per week was recorded for 14 (8 C, 3 SA, 3 SEA), 1 (SEA) and 2 (1 SA, 1 SEA) participants respectively. The participants who reported more than 2 hours of resistance training rated it as being of light-moderate intensity.

There was no difference in the average length of the menstrual cycle, or the day of the menstrual cycle on which the OGTT occurred between the three female groups. Of the total number of 45 women, 10 participants (7 C, 2 SA and 1 SEA) were taking the contraceptive pill.

### Cardiovascular and anthropometric measurements

Groups were similar in their cardiovascular parameters and all participants were normotensive (Table 1). The Asians were shorter and lighter than C, but there was no difference in BMI which was below the value of 25 for C or around 23 for Asians, and is regarded as ‘normal`[29]. There was no difference in waist circumferences which in all groups were well below the classification for central obesity [29]. The waist-hip ratio was higher in female SEA compared with female C, however it was still well below the WHO classification of > 0.85 for central obesity [29]. In females, the percent body fat as measured by bioelectrical impedance (but not by skinfolds) was lower in the SEA (P = .000). For males, subscapular skinfolds were higher in SA and SEA (P = .002) and abdominal skinfolds higher in SA, compared to C (P = .032). Though not significant, suprailliac skinfolds were also higher in SA. However, percent body fat as measured by skinfolds or bioelectrical impedance was not significantly different between the male groups.

**Table 1:** Cardiovascular, anthropometric and body composition characteristics.

	C	SA	SEA	P value
Age (yr)	21 ± 0.4	21 ± 0.4	20 ± 0.3	.320
Birthweight (kg)	3.4 ± 0.1	3.2 ± 0.2	3.3 ± 0.1	.287
RHR (bpm)	72 ± 2	74 ± 2	74 ± 2	.707
SBP (mmHg)	111 ± 2	109 ± 2	105 ± 2	.123
DBP (mmHg)	69 ± 1	69 ± 1	66 ± 1	.158
MAP (mmHg)	83 ± 1	83 ± 1	79 ± 1	.089
Height (cm)	181 ± 2(M)167 ± 2 (F)	174 ± 2*(M)162 ± 2* (F)	172 ± 2*(M)160 ± 1* (F)	**.001.005**
Weight (kg)	76 ± 2(M)59 ± 1(F)	69 ± 2(M)56 ± 2(F)	66 ± 2* (M) 52 ± 1*(F)	**.006.004**
BMI (kg/m^2^)	23 ± 0.6(M)21 ± 0.5(F)	23 ± 0.4(M)21 ± 0.7(F)	22 ± 0.4(M)20 ± 0.4(F)	.593.206
Waist (cm)	77 ± 1(M)69 ± 1(F)	75 ± 1(M)68 ± 2(F)	74 ± 1(M)66 ± 1(F)	.211.199
Hip (cm)	95 ± 2(M)98 ± 1(F)	93 ± 2(M)95 ± 2(F)	91 ± 2(M)88 ± 1* #(F)	.238**.000**
Waist-hip ratio	0.8 ± 0.01(M)0.7 ± 0.01(F)	0.8 ± 0.01(M)0.72 ± 0.01(F)	0.81 ± 0.01(M)0.74 ± 0.01*(F)	.891**.016**
Waist-height ratio	0.42 ± 0.01(M)0.41 ± 0.007(F)	0.43 ± 0.01(M) 0.42 ± 0.008(F)	0.43 ± 0.005(M)0.41 ± 0.005(F)	.732.419
Biceps (mm)	6 ± 1(M)12 ± 2(F)	5 ± 0.5(M)13 ± 1(F)	7 ± 1(M)9 ± 1(F)	.136.111
Triceps (mm)	9 ± 1(M)18 ± 1(F)	11 ± 1(M)20 ± 1(F)	13 ± 1(M) 17 ± 1(F)	.090.296
Subscapular (mm)	11 ± 1(M)12 ± 1(F)	16 ± 1* (M)15 ± 1(F)	15 ± 1* (M)14 ± 1(F)	**.002**.184
Suprailliac (mm)	17 ± 3(M)15 ± 1(F)	24 ± 3(M) 16 ± 1(F)	18 ± 1(M)16 ± 1(F)	.083.797
Abdominal (mm)	15 ± 2(M)18 ± 1(F)	23 ± 2* (M)20 ± 2(F)	17 ± 2(M)18 ± 1(F)	**.032**.373
% BF (skinfolds)	16 ± 1(M) 28 ± 1(F)	22 ± 3(M)30 ± 1(F)	19 ± 1(M)28 ± 0.6(F)	.078.416
% BF (Tanita)	15± 1(M)27 ± 1(F)	17 ± 1(M)27 ± 2(F)	15 ± 1(M)18 ± 1* #(F)	.276**.000**
Day of cycle	17 ± 2	14 ± 2	16 ± 2	.712
Length of cycle (days)	30 ± 1	31 ± 2	33 ± 2	.615

Data are mean ± standard error of the mean (SEM).

* is significantly different from C

# is significantly different from SA, P≤.05, ANOVA, Tukey (post-hoc). RHR: resting heart rate; SBP: systolic blood pressure; DBP: diastolic blood pressure; MAP: mean arterial pressure; BMI: body mass index; %BF: percent body fat; M: male; F: female.

### Nutritional intake

The majority of macronutrient and micronutrient intake of SA and SEA did not differ significantly from C (Table 2). All groups consumed most micronutrients at a higher than recommended daily intake (RDI), with sodium intake amongst the highest, especially for SEA. The percentage RDI of fibre and folate in the diet of SA and SEA, zinc in the diet of SA and calcium in the diet of SEA was significantly lower when compared with that of C. The percentage contribution of monounsaturated fat to the diet of SA was also significantly lower when compared with that of C. The percentage consumption of the macronutrients carbohydrate, fat and protein was as recommended in the three groups.

**Table 2:** Dietary analysis of a 3-day food intake.

	C	SA	SEA	P value
Total energy(kJ)	9441 ± 570	8328 ± 468	9285 ± 773	.389
Carbohydrate (% cont)	48 ± 1	53 ± 2	50 ± 2	.102
Fat (% cont)	33 ± 1	29 ± 1	33 ± 2	.118
Monounsaturated fat (% cont)	13 ± 1	10 ± 0.5*	13 ± 1#	**.023**
Polyunsaturated fat (% cont)	5 ± 0.3	5 ± 0.3	5 ± 0.4	.382
Saturated fat (% cont)	13 ± 1	12 ± 1	12 ± 1	.608
Protein (% cont)	17 ± 0.6	17 ± 0.8	17 ± 0.6	.968
Alcohol (% cont)	1.9 ± 0.7	0.6 ± 0.3	0.3 ± 0.2*	**.030**
Energy (% RDI)	104 ± 11	90 ± 8	93 ± 6	.485
Carbohydrate (% RDI)	89 ± 9	86 ± 8	84 ± 7	.889
Fat (% RDI)	114 ± 13	87 ± 9	103 ± 10	.185
Monounsaturated fat (% RDI)	130 ± 15	90 ± 9	120 ± 12	.063
Polyunsaturated fat (% RDI)	49 ± 6	40 ± 4	48 ± 5	.363
Saturated fat (% RDI)	135 ± 16	106 ± 13	114 ± 13	.325
Protein (% RDI)	194 ± 11	192 ± 20	214 ± 16	.558
Fibre (% RDI)	96 ± 7	73± 5*	76 ± 6*	**.014**
Sodium (% RDI)	344 ± 30	368 ± 37	540 ± 65* **#**	**.007**
Calcium (% RDI)	128 ± 8	108 ± 8	94 ± 10*	**.031**
Folate (% RDI)	191 ± 18	148 ± 10*	143 ± 16*	**.049**
Iron (% RDI)	185 ± 23	148 ± 17	160 ± 21	.437
Magnesium (% RDI)	129 ± 7	109 ± 8	121 ± 13	.319
Niacin (% RDI)	319± 41	273 ± 43	276 ± 21	.607
Phosphorus (% RDI)	178 ± 14	150 ± 13	168 ± 23	.517
Potassium (% RDI)	180 ± 9	143 ± 11	162 ± 14	.091
Riboflavin (% RDI)	203± 32	150 ± 16	132 ± 14	.075
Thiamin (% RDI)	275 ± 47	194 ± 22	184 ± 21	.101
Retinol (% RDI)	160± 12	121 ± 12	163 ± 42	.449
Vitamin C (% RDI)	501 ± 54	392 ± 42	448 ± 66	.378
Zinc (% RDI)	107 ± 8	78 ± 4*	102 ± 11	**.035**

Data are mean ±standard error of the mean (SEM).

*is significantly different from C

# is significantly different from SA, P≤.05, ANOVA, Tukey (post-hoc). LSD (post-hoc used to define differences between groups for folate only). % cont: percent contribution; % RDI: percent recommended daily intake.

### Biochemistry

**Fig 1:**
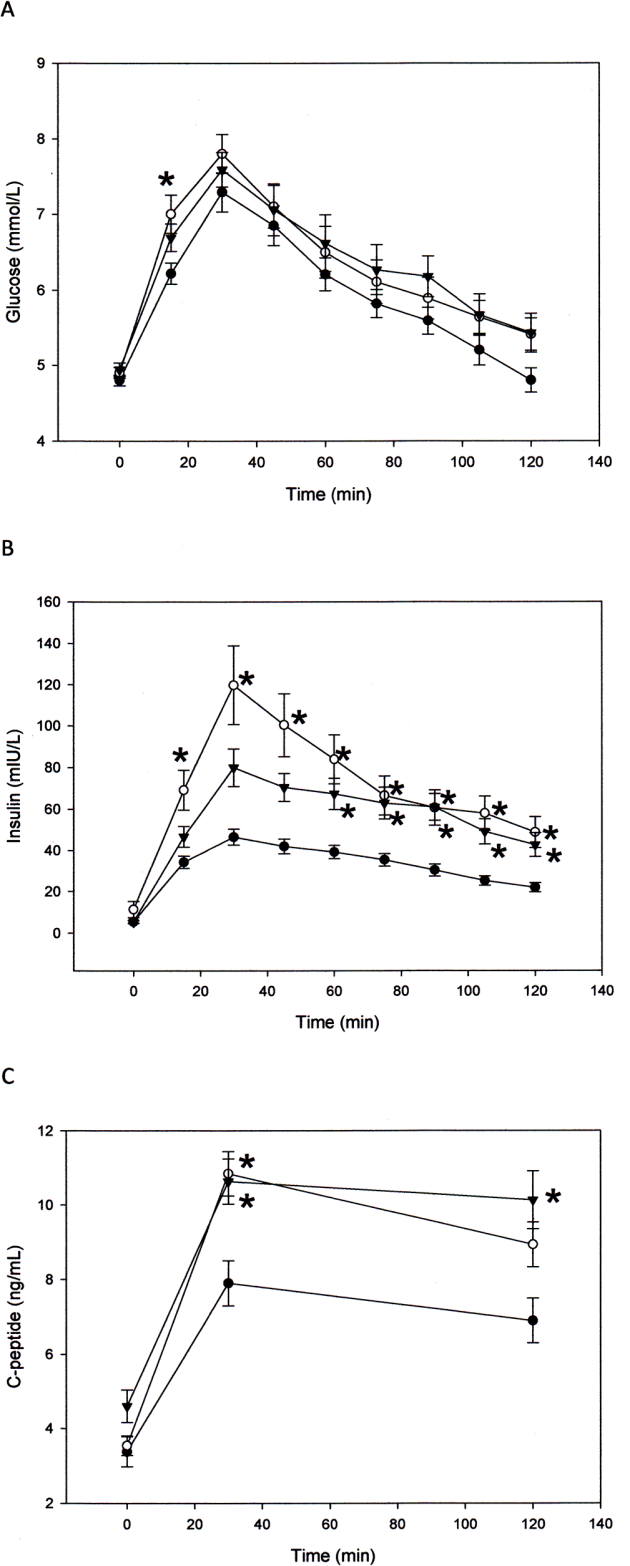
Plasma glucose (A), insulin (B) and C-peptide (C) at baseline and for 2 hrs following an oral glucose challenge in (●) C, (○) SA and (▼) SEA. Data is mean ± standard error of the mean (SEM). * is significantly different from C at same given time point. P≤.05, ANOVA, Tukey (post-hoc).

There was no difference between groups in the average time of collection of the fasting blood sample. Fasting glucose was not significantly different between groups, with all groups displaying normal fasting glucose (Fig 1A) defined as <5.6 mmol/L before meals [30]. Despite this, when compared to C, glucose at 15 min during the OGTT was higher in SA (P = .024). The glucose AUC, however, was not different (Table 3). In addition, blood glucose at 2 hr was well below the impaired glucose tolerance level of > 7.8 mmol/L for all groups (Fig 1A). Fasting insulin, though slightly higher in SA, was also not significantly different (Fig 1B), however, insulin during the OGTT and insulin AUC, was higher in SA and SEA (P < .05, Fig 1B, Table 3). Though not statistically significant, HOMA-IR was also higher in SA (Table 3). There was no correlation between insulin at any time point measured during the OGTT or insulin AUC with height, weight or BMI, when examined for the entire group or separately in the males or females. Fasting C-peptide was not different between groups however C peptide during the glucose challenge rose significantly in the Asian groups compared with C, resulting in a significantly higher C-peptide AUC (Table 3). C-peptide correlated with insulin collected at the same time points of 30 and 120 min (r = .43 and .50, respectively, P < .01). C-peptide AUC also correlated with insulin AUC (r = .46, P < .01). The molar ratio of integrated concentrations of C-peptide to integrated concentrations of insulin were significantly lower in SA, compared with C, indicating a possible decreased hepatic insulin clearance in SA (Table 3). Total cholesterol, triglyceride and LDL-C, while well below the ‘borderline risk’ levels of 5.1, 1.7 and 3.3 mmol/L, respectively, were nevertheless higher in SA (P < .05, Table 3) [30]. Adiponectin was lower in SA (P < .05, Table 3) and a correlation was found between adiponectin and HDL-C (r = .55, P < .01). E selectin was lower in SEA when compared with C (P < .05, Table 3) but no difference was found for any of the other biomarkers studied.

**Table 3:** Biochemical characteristics.

	C	SA	SEA	P value
Glucose AUC(mmol/L)	722 ± 20	765 ± 27	757 ± 29	.447
Insulin AUC(mIU/L)	3988 ± 265	8877 ± 1093*	7002 ± 568*	**.000**
C-peptide AUC(ng/ml)	836 ± 67	1124 ± 53*	1180 ± 53*	**.000**
C-peptide/Insulin (AUC)	11.0 ± 1.0	7.7 ± 0.6*	9.4 ± 0.9	**.045**
HOMA-IR	1.2 ± 0.1	2.3 ± 0.7	1.1 ± 0.1	.093
TC (mmol/l)	3.0 ± 0.2	4.0 ± 0.1*	4.0 ± 0.1*	**.000**
Triglyceride (mmol/l)	0.5 ± .07	0.8 ± 0.1*	0.6 ± 0.1	**.050**
HDL (mmol/l))	1.2 ± 0.05	1.1 ± 0.05	1.4 ± 0.06#	**.016**
LDL (mmol/l)	1.6 ± 0.16	2.3 ± 0.17*	1.6 ± 0.2#	**.009**
TC/HDL	2.4 ± 0.2	3.7 ± 0.2*	2.9 ± 0.1#	**.000**
HbA1C (%)	4.7 ± 0.07	4.9 ± 0.06	5.0 ± 0.06*	**.036**
Microalbumin (mg/l)	11.1 ± 4.0	13.5 ± 5.2	6.0 ± 1.4	.387
Creatinine (mmol/l)	13.6 ± 1.3	13.4 ± 1.5	17.2 ± 4.3	.552
Albumin/Creatinine	0.7 ± 0.3	2.8 ± 1.8	0.4 ± 0.1	.219
Adiponectin (μg/ml)	7.7 ± 0.75	5.4 ± 0.4*	6.0 ± 0.6	**.037**
Leptin (ng/ml)	6.6 ± 1.3	10.8 ± 1.8	7.4 ± 0.8	.086
Cortisol (nmol/l)	1790 ± 32	1778 ± 37	1829 ± 32	.540
ACTH (pmol/l)	6.3 ± 1.6	3.7 ± 0.7	3.2 ± 0.4	.091
NOx (μmol/l)	6.4 ± 1.0	5.5 ± 0.7	5.9 ± 1.0	.812
t-PA (ng/ml)	1.1 ± 0.1	1.3 ± 0.1	1.2 ± 0.1	.631
PAI-1 (ng/ml)	11.9 ± 3.6	12.3 ± 2.4	9.0 ± 1.5	.638
vWF activity (%)	59 ± 5	58 ± 5	50 ± 3	.367
CRP (mg/l)	8.8 ± 4.0	4.0 ± 1.8	3.8 ± 1.3	.332
Homocysteine (μmol/l)	8.9 ± 0.3	10.0 ± 0.6	9.5 ± 0.4	.250
TNF-α (pg/ml)	2.3 ± 0.5	1.4 ± 0.3	3.9 ± 1.1	.062
IL-6 (pg/ml)	2.4 ± 0.3	2.3 ± 0.3	2.7 ± 0.3	.654
VCAM-1 (ng/ml)	904 ± 46	784 ± 50	806 ± 36	.129
E-selectin (ng/ml)	37 ± 4	33 ± 6	21 ± 2*	**.025**

Creatinine and albumin are in urine, all other measurements are in plasma. Data are mean ± standard error of the mean (SEM).

* is significantly different from C

# is significantly different from SA, P < .05, ANOVA, Tukey (post-hoc). AUC: area under curve; C-peptide/insulin AUC: Molar ratio of C-peptide AUC(pmol/L) to Insulin AUC(pmol/L); HOMA-IR: homeostasis model assessment of insulin resistance; TC: total cholesterol; HDL: high density lipoprotein cholesterol; LDL: low density lipoprotein cholesterol; TC/HDL: total cholesterol/high density lipoprotein cholesterol ratio; HbA1C: glycosylated haemoglobin; ACTH: adrenocorticotropic hormone; NOx: nitric oxide metabolites; t-PA: tissue plasminogen activator; PAI-1: plasminogen activator inhibitor-1; vWF: von Willebrand Factor activity; CRP: C-reactive protein; TNF-α: tumor necrosis factor-alpha; IL-6: Interleukin-6; VCAM-1: vascular cell adhesion molecule-1; E selectin: endothelial leukocyte adhesion molecule-1.

All data were also analysed separately for the male and female groups to examine for gender differences. Adiponectin levels remained lower in the male and female SA, though this was not significant when compared with their respective C counterparts. However, a correlation remained between adiponectin and HDL in the female group (r = .53, P < .01). E-selectin remained lower in the SEA of both males and females, but this was significant in the males only with E-selectin levels of 42 ± 7, 29 ± 2, and 21 ± 3 ng/ml in male C, SA and SEA, respectively (P = .011). A correlation was found in the men between total cholesterol and HOMA-IR (r = .51, P < .01), and also for both abdominal (r = .56, P < .01) and suprailliac (r = .58, P < .01) skinfold thickness with fasting insulin.

In females, the leptin levels were higher in SA with 8 ± 1, 16 ± 3, and 10 ± 1 ng/ml in C, SA and SEA, respectively (P = .011). A correlation was found for the women, between leptin and the triceps skinfold (r = .56, P < .01). Leptin also correlated less strongly with the sum of skinfold thicknesss (r = .48), BMI (r = .48), post-meal insulin at 45, 60 and 75 min (r = .46, .45 and .45, respectively) and insulin AUC (r = .41), (each P < .05).

### Genetics

**Fig 2:**
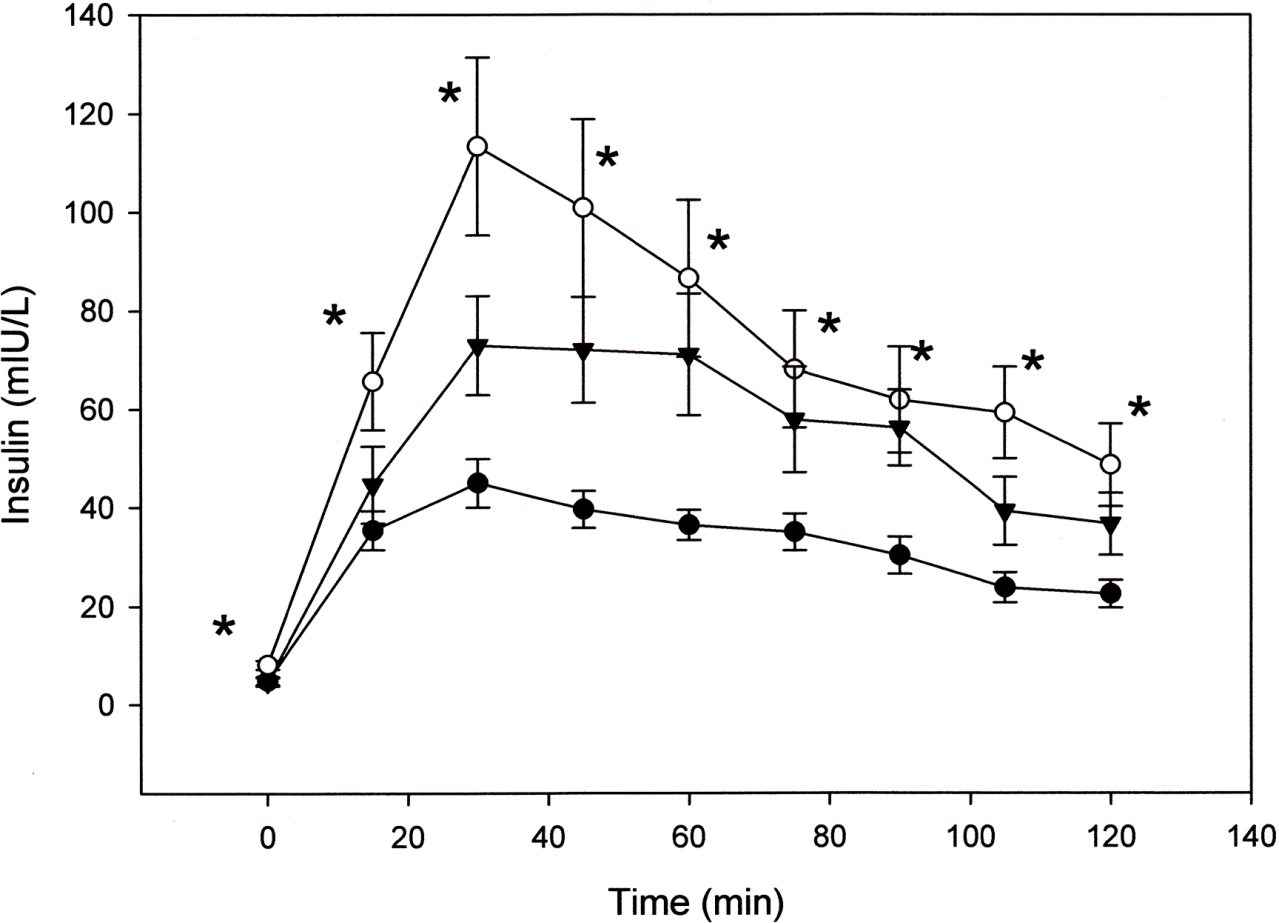
Plasma insulin at baseline and for 2 hrs following an oral glucose challenge in (●) C, (○) SA and (▼) SEA. Data is mean ± standard error of the mean (SEM). * is significantly different from C at same given time point. P≤.05, ANOVA, Tukey (post-hoc).

A total of 22 SNPs in 14 genes selected from the literature as being associated with T2DM [31–47] were tested in 22 C (8 Male (M); 14 Female (F)), 19 SA (9 M, 10 F) and 15 SEA (7 M, 8 F). As this was not an original study aim of the project the sample numbers were restricted by subsequent further ethical approval from the participants following completion of the initial biochemical analysis. This subgroup demonstrated no significant difference in fasting glucose (4.8 ± 0.08, 4.8 ± .09, 4.8 ± .12 mmol/L) and glucose AUC (707 ± 24, 736 ± 34, 734 ± 40 mmol/L) between C, SA and SEA respectively. However insulin following the glucose challenge remained significantly higher throughout the 2 hr period in SA when compared with C (Fig 2). In addition insulin AUC and HOMA–IR were both significantly higher in SA (3892 ± 312, 8758 ± 1260, 6729 ± 840 mIU/L, P = .000, and 1.01 ± 0.16, 1.73 ± 0.19, 0.96 ± 0.21, P = .008, for C, SA and SEA respectively). Association analyses between SNPs and insulin AUC ignoring or including ethnicity, suggested rs7903146, rs12255372 and rs2237892 may be SNPs of interest (Table 4). However allele frequency distributions in C compared with SA indicated a significant difference only in the *MTNR1B* gene for SNPs rs2166706 and rs10830963 (Table 5).

**Table 4:** Association between SNPs and Insulin Area Under Curve (AUC) for 22 Caucasians (C), 19 South Asians (SA) and 15 South East–East Asians (SEA). Analyses were run (1) using a simple association test, ignoring ethnicity, and comparing the entire data set (1 group) with insulin AUC; (2) using an association test including ethnicity as a factor, comparing the 3 different groups (3 levels; C vs SA vs SEA), with insulin AUC; and (3) using an association test including ethnicity as a factor, comparing 2 different groups (2 levels; C vs SA and SEA), with insulin AUC. The data below shows the SNPs for which significance was found. Association analyses were performed using PLINK, P≤.05 indicates significance.

**1**		
**Gene**	**SNP**	**P value**
*TCF7L2*	rs7903146	0.04
*TCF7L2*	rs12255372	0.04
*KCNQ1*	rs2237892	0.09
**2**		
**Gene**	**SNP**	**P value**
*TCF7L2*	rs7903146	0.08
*TCF7L2*	rs12255372	0.24
*KCNQ1*	rs2237892	0.15
**3**		
**Gene**	**SNP**	**P value**
*TCF7L2*	rs7903146	0.21
*TCF7L2*	rs12255372	0.39
*KCNQ1*	rs2237892	0.05

**Table 5:** Allele frequency distributions in Caucasians and South Asians. P≤.05 indicates significant difference, Fisher’s exact test. A significant difference was obtained only in the *MTNR1B* gene for both SNPs studied.

Gene	SNP	Chromosome	Position on Chromosome	Allele frequency Caucasian	Allele frequency South Asian	P value
*PPARG2*	rs1801282	3	12351626	CC: 19 (86%)CG: 3 (14%)	CC: 15 (79%)CG: 4 (21%)	0.68
*PPARG2*	rs3892175	3	12326539	AG: 6 (27%)GG: 16 (73%)	AG: 1 (5%)GG: 18 (95%)	0.1
*TCF7L2*	rs4918789	10	113062048	GG: 2 (9%)GT: 12 (55%)TT: 8 (36%)	GG: 2 (10%)GT: 11 (58%)TT: 6 (32%)	1.0
*TCF7L2*	rs10885409	10	113048313	CC: 2 (9%)CT: 13 (59%)TT: 7 (32%)	CC: 1 (5%)CT: 10 (53%)TT: 8 (42%)	0.89
*TCF7L2*	rs7903146	10	112998590	CC: 11 (50%)CT: 10 (45%)TT: 1 (5%)	CC: 14 (74%)CT: 3 (16%)TT: 2 (10%)	0.12
*TCF7L2*	rs12255372	10	113049143	GG: 12 (54%)GT: 9 (41%)TT: 1 (5%)	GG: 15 (79%)GT: 4 (21%)TT: 0 (0%)	0.19
*TCF7L2*	rs11196205	10	113047288	CC: 2 (9%)GC: 13 (59%)GG: 7 (32%)	CC: 1 (5%)GC: 11 (58%)GG: 7 (37%)	1.0
*TCF7L2*	rs290487	10	113149972	CC: 14 (64%)TC: 8 (36%)TT: 0 (0%)	CC: 10 (53%)TC: 8 (42%)TT: 1 (5%)	0.63
*KCNJ11*	rs5219	11	17388025	CC: 8 (36%)CT: 11 (50%)TT: 3 (14%)	CC: 5 (26%)CT: 13 (69%)TT:1 (5%)	0.53
*FTO*	rs9939609	16	53786615	AA: 4 (18%)AT: 12 (55%)TT: 6 (27%)	AA: 1 (6%)AT: 9 (47%)TT: 9 (47%)	0.34
*CDKAL1*	rs7756992	6	20679478	AA: 11 (50%)AG: 9 (41%)GG: 2 (9%)	AA: 14 (74%)AG: 5 (26%)GG: 0 (0%)	0.25
*HHEX*	rs1111875	10	92703125	AA: 3 (14%)AG: 8 (36%)GG: 11 (50%)	AA: 7 (37%)AG: 9 (47%)GG: 3 (16%)	0.055
*KCNJ153*	rs3746876	21	38299525	CC: 22 (100%)CT: 0 (0%)	CC: 19 (100%)CT: 0 (0%)	1.0
*KCNQ1*	rs2237892	11	2818521	CC: 19 (86%)CT: 1 (5%)TT: 2 (9%)	CC: 19 (100%)CT: 0 (0%)TT: 0 (0%)	0.49
*RAPGEF1*	rs11243444	9	131597867	CC:0 (0%)CT: 3 (14%)TT: 19 (86%)	CC:0 (0%)CT: 6 (32%)TT: 13 (68%)	0.26
*ADIPOQ*	rs182052	3	186842993	AA: 2 (9%)GA: 11 (50%)GG: 9 (41%)	AA: 5 (26%)GA: 6 (32%)GG:8 (42%)	0.29
*ADIPOQ*	rs7649121	3	186850996	AA: 17 (77%)AT: 4 (18%)TT: 1 (5%)	AA: 12 (63%)AT: 4 (21%)TT: 3 (16%)	0.42
*MTNR1B*	rs2166706	11	92958366	CC: 0 (0%)TC: 10 (45%)TT:12 (55%)	CC: 3 (16%)TC: 12 (63%)TT: 4 (21%)	0.02
*MTNR1B*	rs10830963	11	92975544	CC: 15 (68%)GC:7 (32%)GG:0 (0%)	CC: 5 (26%)GC:12 (63%)GG:2 (11%)	0.009
*GCK*	rs4607517	7	44196069	AG: 4 (18%)GG:18 (82%)	AG: 3 (16%)GG:16 (84%)	1.0
*G6PC2*	rs560887	2	168906638	AA: 2 (9%)GA: 9 (41%)GG: 11 (50%)	AA: 0 (0%)GA: 4 (21%)GG: 15 (79%)	0.13
*PCK1*	rs2071023	20	57560878	CC: 5 (23%)GC: 9 (41%)GG: 8 (36%)	CC: 9 (47%)GC: 8 (42%)GG: 2 (11%)	0.098

## Discussion

This study found that the increase in insulin following an oral glucose load was highest in SA compared with C. The relative IR of SA was also reflected by a higher HOMA-IR and non-fasting glucose. These results support a previous finding [4], despite a narrower age range (18–25 years, compared with 18–35 years) and do not appear to be attributed to differences in gender, age, birth weight, BMI, percent body fat, waist circumferences and waist-height ratios, as these variables were similar among the groups. Recently, some researchers have highlighted a confounding factor in the OGTT of a smaller body mass receiving the same 0.75 g bolus dose of glucose as a larger person [48, 49]. However, though the Asians were shorter and lighter than C in the current study, there was no correlation of height, weight and BMI with insulin at any time point during the OGTT, or with insulin AUC. It appears therefore, that the smaller body size of the Asians in the current study did not influence the results obtained. The current study also employed an extensive search for differences in a range of biochemical markers of ED, and found lower levels of adiponectin in the SA group. Increased levels of leptin were also evident in female but not male SA. However the lack of other significant biochemical differences in the markers of ED suggests that higher non-fasting insulin, which is indicative of a reduced insulin sensitivity and carbohydrate tolerance, may precede the development of ED and other features of the metabolic syndrome. It is not surprising perhaps, as these men and women are still quite young and ‘healthy’. Although this is an important first study, further research incorporating a direct measure of ED would be of value.

C-peptide is co-secreted with insulin from pancreatic β-cells, and is considered a reliable marker of pancreatic β-cell function [50]. In the current study, an increase in C-peptide correlated with an increase in insulin. Post meal circulating levels of C-peptide and C-peptide AUC were significantly higher in the two Asian groups suggesting a β-cell hypersecretion of insulin and C-peptide in SA and SEA compared to C. Hepatic clearance of insulin can be estimated with the C-peptide to insulin molar ratio [50]. This study employed integrated AUC responses of C-peptide and insulin to compute the molar ratio instead of the using the more problematic C-peptide to insulin ratio obtained at individual sampling points [51]. The molar ratio was significantly lower in SA compared to C indicating a decreased insulin clearance by the liver in this Asian group. This suggests an additional mechanism for the observed increased circulating post meal insulin in SA and SEA. Further research using more direct methods to examine insulin and C-peptide kinetics is necessary to validate this observation.

A reduced hepatic clearance of insulin has previously been observed in Asian Indians of similar BMI to the SA in the current study [52]. WHO has recommended a lower BMI as desirable in Asians compared with C, with overweight in Asians classified as a BMI higher than 23 [29]. Under this definition, the average BMI for the male SA in this study is borderline ‘normal’. However, it is not the total amount of fat but differences in regional fat distribution that is thought to influence IR [53]. It is believed that upper body and central obesity may expose the liver to higher free fatty acid concentrations, reducing liver clearance of insulin [52, 54]. In the current study, waist circumferences, which are viewed as a more valid measure than BMI for defining central obesity and disease risk, were well below the ethnic specific cut off points in C (≥ 102 cm males, ≥ 88 cm females), and in SA and SEA (≥ 90 cm males, ≥ 80 cm females) as defined by the National Cholesterol Education Program, or in C (≥ 94 cm males, ≥ 80 cm females), and in SA (≥ 90 cm males, ≥ 80 cm females) as defined by the International Diabetes Federation [55]. WHO defines obesity as having a waist-hip ratio > 0.9 but the ratio observed was lower for each group. In addition, there was also no difference in waist-height ratio between the three groups within each gender.

More visceral fat is thought to be an important contributor to the development of T2DM, however a higher subcutaneous abdominal fat in males may also be associated with IR [53]. In the current study, the thicker abdominal (in male SA) and subscapular skinfolds (in male SA and SEA) may have contributed to a reduction in insulin clearance and therefore a higher insulin response following glucose intake. A correlation was found between abdominal and suprailliac skinfolds and fasting insulin in the group of forty-five men. Future studies using DEXA may show further truncal differences in fat deposition.

Leptin is an adipokine, and thought to be involved in the regulation of glucose and fat metabolism; stimulating glucose uptake into skeletal muscle and fatty acid oxidation [26]. Levels in the circulation are known to correlate with body fat and higher leptin levels in obesity seem to be accompanied by leptin resistance [56]. Leptin levels, which reportedly correlate in particular with subcutaneous fat [26], were correlated with the triceps skinfold in the current study. Though not significantly different, this skinfold thickness was nevertheless slightly higher in female SA. A higher level of circulating leptin was also found in SA females which was double of that in C; and there was also a moderate positive correlation between leptin and insulin AUC. The female SA in the current study are considered to be quite lean. It may be that increased leptin in these young SA females is indicative of either sensitivity to slight increases in regional subcutaneous fat, or alternatively, a very early gender-specific abnormality of adipose tissue as a possible contributor to IR which is independent of body fat content. This may only become more apparent in males at a much later stage as higher circulating levels of plasma leptin have been identified in older SA males (average age = 30 years), with a higher BMI (average = 24 kg/m^2^) who were insulin-resistant but in whom T2DM had not yet developed [57]. In that study levels of the adipokine, adiponectin were also reportedly lower in the men, as there is an inverse relationship between plasma concentrations of leptin and adiponectin.

The current study found significantly lower adiponectin levels in the entire group of male and female SA. Lower adiponectin levels have been reported in T2DM, though the reason for this is unclear and are suggested to predict the development of insulin resistance and T2DM in healthy individuals [58]. Adiponectin improves insulin sensitivity and concentrations are also related to lipid metabolism, with circulating levels negatively correlated with triglycerides and LDL-C, and positively correlated with HDL-C [58]. Adiponectin is believed to affect the catabolism rather than the synthesis of HDL-C. In the current study adiponectin was positively correlated with HDL-C and this relationship was maintained within the female group. Additional studies are necessary to examine the metabolic significance of lower levels of adiponectin, its relationship with ED and its possible use as a diagnostic tool to predict the future development of insulin resistance. The only other marker associated with dysfunction of the endothelium to achieve significant difference between groups in the current study was E-selectin. Increased levels may predict the onset of T2DM in people at risk [59]. Surprisingly, it was C which had the highest circulating levels, however the reason for this is unclear.

Interestingly a trend has been recently identified in females (which does not appear to be present in males) for higher levels of not only E-selectin, but also ICAM-1 and PAI-1 in women prior to the development of prediabetes [60]. This was not found in the females of the current study. There was also no evidence of increased C reactive protein or IL-6 at this stage, both of which are considered to be more strongly associated with an increased risk of T2DM in women rather than men [61].

There have been contradictory findings regarding the impact of the menstrual cycle on glucose regulation in normal healthy women, with some studies suggesting a decrease in insulin sensitivity in the luteal phase [19, 21] and other studies suggesting no difference in the menstrual, follicular, midcycle or luteal phases [18, 20]. In the current study, the OGTT was scheduled after the menstrual phase, as a urine sample for the testing of HbA1C was required on the morning of testing. There was no difference between the three ethnic groups in the length of cycle and the day in the cycle on which the OGTT occurred, therefore a change in insulin sensitivity amongst the women due to the menstrual cycle was not a variable in the current study. In addition, a recent systematic review concluded that hormonal contraceptives appear to have no effect on carbohydrate metabolism in healthy, normal weight women who do not have T2DM [62]. Therefore women using oral contraceptives were not excluded as this was not considered to be a confounding factor.

Asian Indians living in rural areas have a 2–3% prevalence of T2DM, while a fourfold increase is experienced in those living in urban areas or who have migrated to Western countries [63] where they have adopted a diet higher in fat and refined carbohydrate, together with a sedentary lifestyle. Regular resistance training increases muscle mass whereas aerobic physical activity uses large muscle groups and promotes the uptake of glucose and fat into the muscle. As a result, both aerobic exercise and/or resistance training improve glycemic control and reduce the risk of T2DM [17, 64, 65]. However, two-thirds (69%) of participants in the current study were sedentary, or participating in less than 1 hour per week of aerobic exercise. The four participants who reported that they participated in > 2 hours of aerobic activity per week demonstrated average resting heart rates rather than lower resting heart rates which are more indicative of aerobic fitness. In addition, only 18% of the participants were engaging in regular resistance training. The two participants in the study who reported training more than 2 hours weekly, described their training intensity as light-moderate. Therefore it is not believed that there are any confounding effects due to exercise on the results of the current study. It is not currently known whether the absence of regular aerobic exercise and/or resistance training might have a greater unfavourable impact on SA and SEA, compared to C. This is an important question and warrants investigation.

The macronutrient intake among the three groups was similar. This is surprising given that differences within groups included length of time spent living in Sydney, residing on- or off-campus with either family or friends, and religious background, each of which may be expected to significantly impact on dietary intake. However all individuals were attending the same large urban university where a Westernised diet is prevalent. However a lower intake of fibre and monounsaturated fat in the diets of SA occurred, which is consistent with a previous report [66]. The intake of fibre in the current study was also significantly lower in the Asian diets. A limitation is that the individual glycemic loads of the meals were not able to be calculated. Despite no significant difference in percentage fat intake, percentage intake of monounsaturated fat was significantly lower in the SA group and may have contributed to the higher circulating total cholesterol, triglycerides and LDL-cholesterol. Total cholesterol correlated positively with HOMA-IR in the males, indicating that a link between the development of dyslipidemia and hyperinsulinemia leading to T2DM and the metabolic syndrome is already evident. Protein intake for all groups was twice the RDI, while sodium intake was five and a half times higher than recommended in SEA, and three and a half times higher than recommended in SA and C. It has recently been reported that diets high in protein are associated with an increased risk of T2DM [67], while most patients with T2DM have a dietary salt-induced exacerbation of hypertension [68]. Zinc was also lower in the SA diets. It is unknown whether a higher than recommended protein or salt intake, or lower intake of trace elements has a greater adverse effect on Asian compared to Caucasian populations. Ethnic specific dietary guidelines regarding nutritional intake for populations which are more ‘at risk’ of T2DM need to be established.

However, while dietary factors and sedentary lifestyle are thought to have a key influence on insulin resistance and T2DM in SA, genetic factors are also thought to be important [69]. Genome-wide association studies have identified common genetic variation around a number of genes which are thought to influence glucose levels. Of the twenty-two SNPs examined in the current study, it is interesting that only two SNPs, rs10830963 and rs2166706, had a significant difference in allele frequency distribution in C and SA. These SNPs are both near the melatonin receptor MTNR1B. The results of the current study support previous studies that have documented an association of rs10830963 and rs2166706 with an increased risk of T2D among SA [32, 70].

Circulating melatonin is produced by the pineal gland in the brain and is an important signalling molecule in the entrainment of biological rhythms in the body. The main control is the brain’s suprachiasmatic nucleus (SCN), however peripheral clocks are believed to influence and are also influenced by the SCN [71, 72, 73]. Peripheral clocks are found in many organs throughout the body, including the pancreas, liver, adipose tissue and skeletal muscle, all of which are of particular relevance to T2D. A disturbance in the biological clock is present in shift workers or those who suffer from sleep apnoea, and these populations have been identified to be at an increased risk for T2D, with a disturbance in the circadian rhythm affecting glucose homeostasis [71, 72, 73].

In humans there are two functional melatonin G protein coupled receptors, *MTNR1A* (MT_1_) and *MTNR1B* (MT_2_), though a third possible melatonin receptor has been identified [74]. MT_2_ receptors are in various tissues including adipocytes, liver, skeletal muscle and pancreatic β-cells [73, 75, 76]. In pancreatic β-cells melatonin appears to alter insulin concentrations via three separate pathways. The predominant action of melatonin at the MT_2_ receptor is to lower cyclic adenosine monophosphate (cAMP), which subsequently decreases insulin secretion [73, 76]. Melatonin at the MT_2_ receptor can also inhibit cyclic guanosine monophosphate (cGMP) which also inhibits insulin secretion. In contrast, melatonin can induce insulin secretion by stimulating the IP3-signalling pathway [73, 76]. However, as the predominant action of melatonin appears to be to decrease insulin release from the pancreas, it has been suggested that melatonin protects β-cells from functional overstrain and exhaustion [74, 77]. Reduced melatonin is linked to an increased risk of T2D, and indeed nocturnal melatonin levels are lower in T2D patients and in diabetic rat animal models [78, 79]. Catecholamines have been implicated as key to explaining the insulin-melatonin balance as they trigger melatonin synthesis and inhibit insulin secretion [80]. Indeed in the early stage of T2D, rats exhibit increased circulating insulin together with diminished catecholamine and melatonin levels.

Additional research supporting the involvement of melatonin in glucose homeostasis includes the observation that melatonin increases glucose uptake into skeletal muscle and adipose tissue, and decreases nocturnal glucose production by the liver [81]. Insulinemia in diabetic rat strains is reversed with melatonin treatment [77]. Removal of melatonin in rats by pinealectomy decreases GLUT4 in adipose tissue and muscle leading to glucose intolerance and insulin resistance which is restored by administration of melatonin [77]. Receptor knockout mice for the MT_2_ receptor exhibit disturbances in circadian rhythm, higher levels of insulin and impaired glucose homeostasis [75]. The vast majority of research has been carried out in mice and rats, which are nocturnal animals and care needs to be taken when extrapolating data to humans. However recent human genome-wide association studies have provided further insight into the relationship between the MT_2_ receptor and T2D.

To date, various authors have identified seven SNPs located near or inside the gene encoding *MTNR1B* with an association with T2D in Asian (Indian, Sri Lankan, Chinese, Korean, Japanese) and European ethnicities [32, 70, 75, 82, 83, 84]. Of the seven SNPs, rs10830963 appears to be the most strongly associated with an increase in fasting plasma glucose, glucose AUC and HbA1C; and a decrease in pancreatic β-cell function, basal insulin secretion and plasma insulin [75, 85]. It appears to affect β-cell function directly and is associated with a defective early insulin response and a decreased β-cell glucose sensitivity [44, 86, 87, 88].

The rs10830963 G-allele appears to have a greater risk on the transition from normal glucose tolerance to prediabetes than on prediabetes to T2D and is thought to be an important influence on glucose levels from childhood onwards [89]. It has been reported that each G allele in rs10830963 is associated with an increase of 0.07 mmol per litre in fasting glucose levels [90]. This allele is also associated with gestational diabetes [75]. Individuals older than 45 years of age who are carrying the rs10830963 G allele, show a higher expression of *MTNR1B* in pancreatic islets [87]. This has been reported in diabetic rats as well as diabetic humans [75]. It is not known whether this is a physiological adaptive response to reduced melatonin levels or whether it is part of the pathology of T2D. It has been proposed that an increase in MT_2_ receptor expression could increase the inhibitory downstream signalling leading to an overall decrease in insulin release in T2D [75, 87].

The rs2166706 variant has also been associated with an increase in fasting plasma glucose and HbA1C and a decrease in pancreatic β-cell function [75]. At a molecular level, it is not known what functional relevance rs10830963 or rs2166706 have on the receptor protein with rs10830963 located inside the only intron of *MTNR1B*, and rs21667016 in the 11kb region upstream of the gene. The rs10830963 variant does not appear to disrupt consensus transcription factor binding or cryptic alternative splice sites [90]. Recently, a large-scale exon resequencing of two exons of *MTNR1B* has examined 40 rare mutants and demonstrated impairment in melatonin binding and signalling, establishing a functional link between this receptor and T2D risk [91]. Further research on rs10830963 and rs2166706, and other gene variants is required to determine their effect on the expression of, or the function of the MT_2_ receptor. Additionally, clinical trials examining the therapeutic benefit of melatonin in human T2D needs to be investigated, particularly in ethnicities such as SA where T2D is prevalent.

The investigation of SNPs was not an original study aim of the current project. As a result, a limitation of the current study is that no data exist on the cohort’s sleep habits; for example, duration of sleep, exposure to light immediately prior to and during sleep, awakening during the night, and work habits such as night shifts. It is anticipated that the cohort of young university students would have quite varied sleep patterns and certainly future studies in a similar group should include this information.

In summary, it is evident that young SA and SEA are at a greater risk of developing T2DM, cardiovascular disease and the metabolic syndrome. This study confirms that early identification of young non-obese ‘at risk’ individuals through the employment of the OGTT and the measurement of non-fasting insulin is more effective than fasting glucose alone. A decreased hepatic clearance of insulin may be contributing to the hyperinsulinemia following an oral glucose load. Higher non fasting insulin appears to precede the development of ED and other features of the metabolic syndrome. Further research regarding specific dietary and exercise intervention which may modify the OGTT response in these young Asians to more closely mimic their C counterparts is required. In addition to environmental influences, one possible genetic contribution in SA may be variation near the melatonin receptor *MTNR1B*. The current study provides further impetus for future research in the role of circadian rhythms and melatonin signalling in the development of T2DM.
